# When Two Worlds Collide: Navigating Diabetes in Cystic Fibrosis

**DOI:** 10.7759/cureus.84110

**Published:** 2025-05-14

**Authors:** Wisal Ahmad, Jansher Khan, Ahmad J Hashmat, Anusha Khan, Aftab Ahmad, Jalal Dildar, Meenal Sikander, Sabeena Ahmad, Shayan Zakir, Syed S Raza, Saira K Awan, Giustino Varrassi

**Affiliations:** 1 Internal Medicine, Glangwili General Hospital, Carmarthen, GBR; 2 Medical Education and Simulation, Postgraduate Medical Institute, Peshawar, PAK; 3 Internal Medicine, Richmond University Medical Center, Staten Island, USA; 4 Medical Ethics, Northwell Health, Long Island Jewish Medical Center, New Hyde Park, USA; 5 General Medicine, Cork University Hospital, Cork, IRL; 6 Nephrology, Richmond University Medical Center, Staten Island, USA; 7 Medicine, Rehman Medical Institute, Peshawar, PAK; 8 Oncology/Hematology, University of Virginia Medical Center, Charlottesville, USA; 9 Anesthesiology, University of Virginia Medical Center, Charlottesville, USA; 10 Physiology, Khyber Medical College/Khyber Teaching Hospital, Peshawar, PAK; 11 Internal Medicine, Community Medical Centers, Fresno, USA; 12 Neurology, Advanced Neurology P.C., Brooklyn, USA; 13 Medicine, Kabir Medical College, Peshawar, PAK; 14 Pain Medicine, Fondazione Paolo Procacci, Rome, ITA

**Keywords:** cystic fibrosis (cf), cystic fibrosis-related diabetes, diabetes and endocrinology, glucagon-like peptide 1 (glp-1), insulin treatment

## Abstract

Cystic fibrosis (CF) is an autosomal recessive genetic disorder characterized by the dysfunction of the cystic fibrosis transmembrane conductance regulator (CFTR) gene on the long (q) arm of chromosome number 7. It is characterized by the buildup of thick, sticky mucus that can damage various body organs. The most commonly affected organs are the pancreas, liver, intestines, and lungs. Diabetes mellitus is a complex multi-system metabolic disorder. It is characterized by the relative or absolute insufficiency of insulin secretion with or without concomitant insulin resistance, leading to high blood sugar levels. One of the feared complications of CF is cystic fibrosis-related diabetes (CFRD). In this narrative review, we examine various treatment options, their mechanisms of action, their side effects, and their impact on the lives of patients with CFRD. In conclusion, insulin remains the cornerstone of treatment in the management of CFRD. However, oral medications for diabetes can also be considered safe and effective, in selected patients, with stable liver function and preserved lung capacity.

## Introduction and background

Cystic fibrosis (CF) is an autosomal recessive condition. It is commonly inherited in the Caucasian population and is estimated to affect one in 2500 live births. Early screening, diagnosis, and prompt interventions have led to an improved median survival age, currently reaching approximately 45 years [1]. The disease process of CF affects glucose metabolism in a number of ways that are known under the umbrella term cystic fibrosis-related diabetes (CFRD).

CFRD begins with insulin deficiency and eventually results in a swift decline of lung function, an inability to gain weight, and eventually metabolic failure. CFRD has a 15-30% prevalence in patients affected by CF. Thirty-three percent are symptomatic diabetics. In spite of modern-day advances in the field of medicine, the progression of CFRD cannot be completely blocked [2]. Patients with CFRD have on average six times higher mortality rates in comparison to those with CF only. CFRD patients have a median age of 35.6 years [3].

The pathogenesis of CFRD is the progressive pancreatic tissue destruction that ends in beta cell dysfunction, leading to lower-than-normal levels of insulin hormone. An oral glucose tolerance testing (OGTT) is recommended to diagnose CFRD. Adverse outcomes have already occurred prior to the diagnosis at different stages of dysglycemia.

In order to improve the screening tools and prevent debilitating complications caused by dysglycemia, a two-hour 75-gram OGTT screening is recommended for CFRD [4,5]. Hemoglobin A1c (HbA1c) testing has also been suggested. HbA1c ≥5.8% is a compelling tool for CFRD screening and reduces the need for OGTT by 50.7% [6].

Insulin therapy remains the cornerstone of treatment [5]. Oral medications, including metformin, sulfonylureas, and thiazolidinediones, are also warranted. This narrative review will be mainly focused on the different treatment modalities, their mechanisms of action, their side effects, and their impact on the lives of CFRD patients.

## Review

Methodology

This narrative review employs a comprehensive approach to gather, evaluate, and synthesize relevant literature. 

Literature Search Strategy

To narratively review diabetes in CF, a comprehensive literature search was conducted using multiple databases, including PubMed, Scopus, Web of Science, and Google Scholar.

The search queries were constructed using a combination of Medical Subject Headings (MeSH) terms and keywords. The following strategy was employed to ensure an exhaustive collection of relevant studies. The search was based on the following key terms, which were combined using Boolean operators (AND, OR) to capture studies from a range of disciplines: "cystic fibrosis", "management", "treatment", and "diabetes". The literature search was limited to study types such as peer-reviewed original research articles, systematic reviews, meta-analyses, and clinical trials. Studies published in only English were selected. This was done using strategies developed through consulting team members and published literature on the subject of biomedical reviews using the Scale for the Assessment of Narrative Review Articles (SANRA) scale [7]. The synthesized findings were critically evaluated, analyzed, and then presented narratively. Papers that defined the relationship between CF, CFRD, and treatment options were evaluated.

Ethical Considerations

Ethical approval was unnecessary as the study involved analyzing preexisting published data. This narrative review adheres to ethical principles and guidelines for conducting narrative studies. No primary data collection from human subjects was performed for this review; therefore, ethical approval was not required.

Pathogenesis of CF

Pathophysiology of CFRD

CF has an interesting disease course. It is a monogenic disease with numerous phenotypic fluctuations [8-10]. Patients can exhibit a wide range of clinical symptoms, even if they have inherited similar genotypes. The clinical course of CF can vary and include sinusitis, congenital bilateral absence of the vas deferens (men), progressive lung disease, exocrine pancreatic insufficiency resulting in metabolic failure, malabsorption of nutrients, impaired growth, irregularities in glucose metabolism, and the development of full-fledged diabetes (CFRD) [11-14].

The pathophysiology of cystic fibrosis transmembrane conductance regulator (CFTR) deficiency and dysfunction can be described by the normal functioning of CFTR and what patients lack [15-19]. CFTR works as a chloride channel. It facilitates the transfer of ions across apical membranes of the epithelial cells in the body. Other key functions that the CFTR serves are bicarbonate secretion and the inhibition of sodium transport [20-24]. Therefore, mutations leading to CFTR dysfunction have dire consequences, which are associated with a phenotype characterized by both lung disease and exocrine pancreatic insufficiency [25,26].

It is worth noting here that CFRD and type 1 diabetes (DM1) are primarily problems with a decrease in insulin production. This autoimmune nature of pathogenesis seen in DM1 is, however, not seen in CFRD. CF patients with or without CFRD do not show any difference in the frequency of auto-antibodies [27,28].

The pathophysiology of CFRD has been presented in a tabulated form (Table 1).

**Table 1:** Pathophysiology of CFRD GLP-1: glucagon-like peptide 1; GIP: gastric inhibitory peptide; PERT: pancreatic enzyme replacement therapy; TCF7L2: transcription factor 7-like 2; CFRD: cystic fibrosis-related diabetes This table was created by the authors based on [29,30].

Feature	Characteristic
Islets	Destruction of islets
Amyloid	Present
Insulin status	Partial deficiency of insulin
GLP-1	Improved secretion with PERT
GIP	Secretion normal but efficacy decreased
Hyperglucagonemia	Absent
Genetic predisposition	TCF7L2

Pathophysiology of Diabetes

The pathophysiology of diabetes involves consistently high blood glucose levels. It can be due to a complete lack of insulin secretion from the pancreas (DM1), insulin resistance, a lack of insulin sensitivity, and an inadequate compensatory response by the pancreas (type 2 diabetes) or a combination of exocrine pancreas abnormalities (type 3C diabetes). Chronic pancreatitis, CF, hemochromatosis, and pancreatic ductal adenocarcinoma are the most common causes of type 3C diabetes [31,32].

History and physical

A larger number of patients with CFRD do not show any symptoms at diagnosis. However, the most common symptoms of diabetes, such as frequent urination (polyuria) and excessive thirst (polydipsia), may occur less frequently in CFRD patients. Weight loss despite good nutrition is usually the first symptom of CFRD. Therefore, weight monitoring and nutritional assessment are pivotal in pediatric CF patients.

Role of CFTR gene in the pancreas

The pancreas is a dual-function organ. It acts as both an exocrine and endocrine gland. The function of the exocrine pancreas is to secrete approximately 2 liters per day of isotonic, bicarbonate-rich fluid and digestive enzymes. This is known as pancreatic juice. The exocrine pancreas can be further divided into acinar and ductal cells, which are two types of epithelial cells. By volume, 90% of the pancreatic gland is acinar cells, while the remaining 10% consists of ductal cells, mucus-secreting cells, and endocrine cells.

The apical membrane of ductal cells exhibits a significantly higher expression of CFTR compared to the acinar cells. The ductal cells form a tubular network throughout the pancreatic gland. Thus, the expression of CFTR in ductal cells is crucial for electrolyte and fluid secretion from the gland. This helps to understand the disease process of CF, in which CFTR-dependent fluid secretion is nearly nonexistent [33].

Studies suggest that insufficient ductal bicarbonate and fluid secretion may lead to the destruction of the pancreatic gland. One of the consequences of inadequate bicarbonate secretion is pancreatic juice with an acidic pH, which is less than 6.5 (normal: 7.5-8.0) [34]. The acidic pancreatic juice leads to an increase in mucus viscosity and decreases the solubility of secreted digestive enzymes. Consequently, mucin and protein plugs form within the ducts of the pancreatic gland. This also activates the digestive enzymes prematurely. Clinically, it manifests as the destruction of the pancreatic gland, which is one of the hallmark features of CF. Thus, we can confidently say that CFTR plays a crucial role in bicarbonate and fluid secretion in the pancreas [35].

Treatment of CFRD

CFRD is unique and can be treated with various pharmacological options. In the following subheadings, we will explore the different options available for managing blood glucose levels, assess their effectiveness, examine their mechanisms of action, and discuss their major side effects.

Insulin 

Since the pathophysiology of CFRD involves pancreas destruction, the best option is to replace insulin exogenously [14]. Insulin not only improves blood sugar levels but also reduces episodes of lung exacerbation, thereby reducing morbidity, enhancing the quality of life, and decreasing overall mortality in this patient population. It is observed that CF has already damaged the pancreatic gland before the symptoms of diabetes appear. The classic symptoms of diabetes, polyuria, polydipsia, and polyphagia, are only observed in one-third of the CFRD patient population. Therefore, physicians should have a high level of suspicion for CFRD and begin replacing insulin early in the disease course.

The usual surrogate endpoints, such as HbA1c and OGTT, cannot be solely relied upon for CFRD patients due to the complexity of the disease process. Frequently used insulin regimens include monotherapy with a long-acting insulin or a bolus-only regimen. The basal-bolus insulin regimen, which involves administering long-acting insulin once or twice a day, along with a rapidly acting insulin before each meal, offers the most effective glycemic control [15].

It is worth noting that once there is significant lung damage, denoted by a forced expiratory volume in one second (FEV1) of less than 60% (normal FEV1 range is within 80% of the reference value), insulin is the best available option. One of the major impediments in using insulin is that it is only available in injectable form. This limitation contributes to poor patient compliance, the stigma associated with using needles, and frequent incidents of hypoglycemia [24].

Oral Anti-diabetic Medicines

The most common oral anti-diabetic medications are metformin, sulfonylureas, and thiazolidinediones. CF is a complex disease that affects multiple organs, impairing their optimal functioning. Thus, this should be considered before prescribing oral medications to control blood glucose levels [10]. Not everything is bad in the world of oral anti-diabetic medications. They also carry some benefits that cannot be ignored. Oral medicines are easy to take, providing a pain-free experience and promoting good patient compliance.

Oral medications, such as metformin, can lower blood glucose levels while also possessing anti-inflammatory and insulin-sensitizing properties. Thus, metformin can be initially prescribed to patients as an outpatient oral medication, with the close monitoring of kidney and liver function. Since metformin increases the risk of lactic acidosis, it should never be given to patients with an advanced disease process or those who suffer from frequent episodes of hypoxia [12].

In patients with stable liver function, sulfonylureas and thiazolidinediones can be administered on an outpatient basis to manage CFRD after initially stabilizing spiking blood sugar levels with insulin. Thiazolidinediones carry an additional risk of liver injury, and patients should be monitored for hepatic toxicity [13].

Hypoglycemia is a common occurrence when using sulfonylureas [24]. The sulfonylureas work by enhancing insulin release from the pancreas and may also decrease beta-cell mass. Since the pancreatic gland is already damaged by CF, prescribing sulfonylureas for CFRD can further worsen the condition (Table 2). 

**Table 2:** Drug to be avoided or used with caution in high-risk group CF: cystic fibrosis

Drug	High-risk group (medical condition)
Metformin	Advances stages of CF, preexisting liver disease
Sulfonylureas	Contraindicated in CF
Thiazolidinediones	Liver and kidney disease

CFRD Management Guideline

The Cystic Fibrosis Foundation (CFF) and American Diabetes Association (ADA) have published guidelines for the management of CFRD patients. These were endorsed by the Pediatric Endocrine Society. These guidelines serve as a crucial tool for screening, diagnosing, and managing CFRD. The complete summary of recommendations for all individuals with diabetes can be found in the ADA standards of medical care. These are published annually in the January supplement of Diabetes Care [36]. We have presented the management guidelines in a tabulated form (Table 3). Furthermore, therapy with newer drugs, such as elexacaftor, tezacaftor, and ivacaftor, has shown promising results with improved glucose regulation in some patients [36].

**Table 3:** Summary of CFRD management guideline SMBG: self-monitoring blood glucose; ADA: American Diabetes Association; HbA1c: hemoglobin A1c; CFRD: cystic fibrosis-related diabetes This table was created by the authors based on [37].

CFRD management
Patients should be treated with insulin therapy
Patients on insulin should perform SMBG at least 3× daily
Patients should strive to attain plasma glucose goals as per ADA recommendations for all people with diabetes
HbA1c goal <7% and measured every three months
Moderate aerobic exercise for at least 150 minutes/week
Complication monitoring: (1) Hypoglycemia education. (2) Annual monitoring for microvascular complications beginning 5 years after CFRD diagnosis. (3) An annual lipid profile for patients with any of the following risk factors: obesity, family history of coronary artery disease, or immunosuppressive therapy following lung transplantation

Complications in CFRD patients

In addition to the CF complications, CFRD patients experience diabetic and additional complications. We have presented these in the form of a table in this narrative review (Table 4).

**Table 4:** Summary of complications seen in patients with CFRD FEV1: forced expiratory volume in one second; FVC: forced vital capacity; CFRD: cystic fibrosis-related diabetes This table was created by the authors based on [38-41].

CFRD complications
Compromised nutritional status
Compromised lung function: reductions in FEV1and FVC
Reduced insulin production
Excessive protein catabolism
Susceptibility to respiratory infections
Autonomic neuropathy: diminished cardiorespiratory reflexes

Vascular Complications in CFRD

Vascular damage is a key feature of diabetes. The lungs are rich in extensive capillary network. Studies have found substantial evidence of damage to pulmonary microvasculature in diabetes. This is present along with nephropathy and retinopathy [38,42], pointing towards the concept of "diabetic lung" [43,44]. The large microvascular reserve in the pulmonary tissue ensures that these lung changes in non-CF diabetic patients are subclinical.

Challenges and future directions

CFRD is a challenging and potentially life-threatening medical condition that requires a comprehensive understanding to effectively manage its symptoms and potential complications. Patient and parental education on understanding the disease is a very challenging aspect, as they often lack essential knowledge. This results in unintentional, yet poor, treatment compliance. Education must involve empowering patients with knowledge about the disease. As patients are now becoming active partners, it is necessary to develop educational programs tailored to the needs of CF patients [37].

Limitations and recommendations

There are a few limitations to this review. Firstly, there was very little data available on oral anti-diabetic medicines, and only a few randomized controlled trials (RCTs) have been conducted so far. Secondly, we only included articles that were published in the English language. Studies that were published in other languages were not included in the literature review for this study. It is recommended that more RCTs be conducted to explore different treatment options and identify the best regimens based on evidence supported by published literature. Additionally, efforts should be made to develop novel methods for improving CFRD patients' quality of life.

## Conclusions

The management of CFRD requires a nuanced approach due to the complex interplay between the nutritional demands of CF and the metabolic challenges of diabetes. Insulin therapy remains the cornerstone of treatment, especially in patients with advanced lung disease (FEV1 <60%) or when rapid glycemic control is necessary. Oral anti-diabetic medications can be considered in selected patients with stable liver function and preserved lung capacity. Medications such as metformin, sulfonylureas, and thiazolidinediones may offer additional benefits by improving insulin sensitivity and reducing inflammation, contingent upon renal, hepatic, and pulmonary functions being closely monitored. After all, treatment plans must be individualized, balancing the need for adequate caloric intake, glucose control, and the unique comorbidities associated with CF. This will ensure both metabolic stability and quality of life in the vulnerable population.
